# Cyst Type Differentiates Rathke Cleft Cysts From Cystic Pituitary Adenomas

**DOI:** 10.3389/fonc.2021.778824

**Published:** 2021-12-10

**Authors:** Sherwin Tavakol, Michael P. Catalino, David J. Cote, Xian Boles, Edward R. Laws, Wenya Linda Bi

**Affiliations:** ^1^ Department of Neurosurgery, Brigham and Women’s Hospital, Harvard Medical School, Boston, MA, United States; ^2^ Department of Neurosurgery, University of Oklahoma Health Sciences Center, Oklahoma City, OK, United States; ^3^ Department of Neurosurgery, University of North Carolina Hospitals, Chapel Hill, NC, United States; ^4^ Department of Neurosurgery, Keck School of Medicine of USC, Los Angeles, CA, United States

**Keywords:** cystic sellar lesion, pituitary adenoma, pituitary cyst, pituitary tumor, Rathke cleft cyst, craniopharyngioma

## Abstract

**Purpose:**

A classification system for cystic sellar lesions does not exist. We propose a novel classification scheme for these lesions based on the heterogeneity of the cyst wall/contents and the presence of a solid component on imaging.

**Methods:**

We retrospectively reviewed 205 patients’ medical records (2008–2020) who underwent primary surgery for a cystic sellar lesion. Cysts were classified *a priori* into 1 of 4 cyst types based on the heterogeneity of the cyst wall/contents and the presence of a solid component imaging. There was high interrater reliability. Univariable and multivariable models were used to estimate the ability of cyst type to predict the two most common diagnoses: Rathke cleft cyst (RCC) and cystic pituitary adenoma.

**Results:**

The frequencies of RCC and cystic pituitary adenoma in our cohort were 45.4% and 36.4%, respectively. Non-neoplastic lesions (e.g., arachnoid cysts and RCC) were more likely to be Type 1 or 2, whereas cystic neoplasms (e.g., pituitary adenomas and craniopharyngiomas) were more likely to be Type 3 or 4 (p<0.0001). Higher cyst types, compared to Type 1, had higher odds of being cystic pituitary adenomas compared to RCCs (OR: 23.7, p=0.033, and 342.6, p <0.0001, for Types 2 and 4, respectively). Lesions with a fluid-fluid level on preoperative MRI also had higher odds of being pituitary adenomas (OR: 12.7; p=0.023). Cystic pituitary adenomas were more common in patients with obesity (OR: 5.0, p=0.003) or symptomatic hyperprolactinemia (OR: 11.5; p<0.001, respectively). The multivariable model had a positive predictive value of 82.2% and negative predictive value of 86.4%.

**Conclusion:**

When applied to the diagnosis of RCC versus cystic pituitary adenoma, higher cystic lesion types (Type 2 & 4), presence of fluid-fluid level, symptomatic hyperprolactinemia, and obesity were predictors of cystic pituitary adenoma. Further validation is needed, but this classification scheme may prove to be a useful tool for the management of patients with common sellar pathology.

## Introduction

Cystic lesions in the sellar region encompass a broad gamut of pathologies, which can be challenging to distinguish on imaging, given their variability in clinical presentation and heterogeneity of cyst appearance. The presence of a sellar cyst generates a range of presumptive diagnoses — with pathologies including cystic pituitary adenomas, craniopharyngiomas, Rathke cleft cysts (RCC), arachnoid cysts, epidermoid cysts, dermoid cysts, and xanthogranulomas, among others (1, 2). Although some cystic lesions are benign, incidentally found lesions, others can lead to significant morbidity through disruption of hormonal axes or associated mass effect. Incidentally found lesions such as small pituitary adenoma or RCC may be observed, while surgical resection is indicated for symptomatic lesions or those requiring tissue diagnosis. Clinical and imaging factors are often used to generate a preliminary diagnosis and guide management. This variation in treatment pathways necessitates further study into the features that distinguish these various cystic pathologies, especially the two most common pathologies: RCC and cystic pituitary adenoma. No validated classification system of cystic sellar lesions exists. The localization of the lesion within the sella has been classically used to differentiate lesions originating from the pars intermedia (e.g., RCC) from those originating from the pars distalis (e.g., classic adenomas). Anterior lesions from the pars distalis often exhibit posterior displacement of the infundibulum, and posterior lesions from the pars intermedia often displace the infundibulum anteriorly. For large lesions extending into the suprasellar cisterns, the infundibulum is often hard to identify, and, therefore, this method cannot reliably predict the origin of the lesion. In this study, the classification scheme can be applied to every lesion, regardless of size using the heterogeneity of the cyst wall/contents and presence of a solid component on preoperative magnetic resonance imaging (MRI). We used this *a priori* classification scheme and applied it to a retrospective cohort of patients.

## Materials And Methods

We retrospectively queried an institutional neurosurgical database from April 2008 to January 2020 for cases with the keywords “cyst” and “cystic” in preoperative radiology reports. Only patients with a final tissue diagnosis after surgical excision and/or cyst drainage were included in the study. Patients with non-sellar cysts, such as pineal cyst or epidermal inclusion cyst, were excluded. This study was approved by our institutional review board for ethical conduct of research and protection of human subjects. Patient consent was not required since no identifying information was used in this article.

Our primary aim was to apply an *a priori* cyst classification scheme and compare cystic lesion type with final pathologic diagnosis. Pre-operative thin-slice MRI (1.5T or 3T) and clinical and biochemical data were reviewed for each case. T1 pre- and post-gadolinium contrast images were reviewed along with T2-weighted images. The cyst classification scheme was developed based on focused discussions amongst the authors prior to its application to cyst classification in a retrospective fashion. Classification was independently performed by three authors (ST, MPC, and DJC) who were blinded from the final cyst pathology. Agreement by two or more authors was required for final cyst classification. Inter-rater reliability was assessed using Fleiss’ kappa (κ). Confirmed lesion identity was extracted from pathology reports. Fisher’s exact test was used to evaluate the relationship between cystic lesion type and lesion pathology.

**Figure 1:**
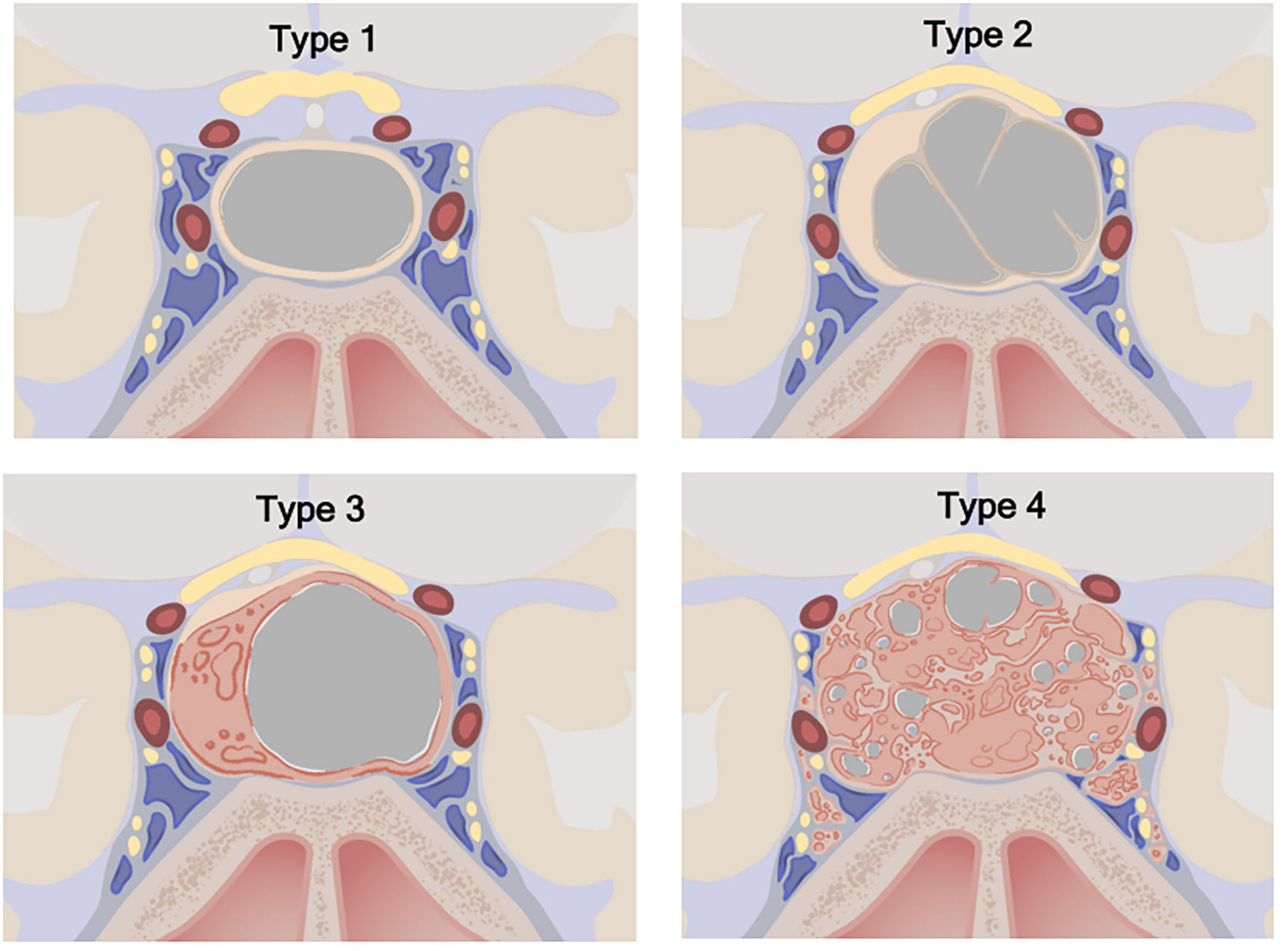
Cystic sellar lesion classification scheme. Type 1: No solid component; well-circumscribed, homogenous cyst. Type 2: Little or no solid component; irregular cyst, with septations or abnormal walls. Type 3: Obvious solid component; well-circumscribed homogenous cyst. Type 4: Obvious solid component present; irregular cyst(s) with septations or abnormal walls. Type 1: n=68 (33.2%), Type 2: n=72 (35.1%), Type 3: n=10 (4.9%), Type 4: n=55 (26.8%). ©2020 Xian Boles, Used by permission.

**Figure 2:**
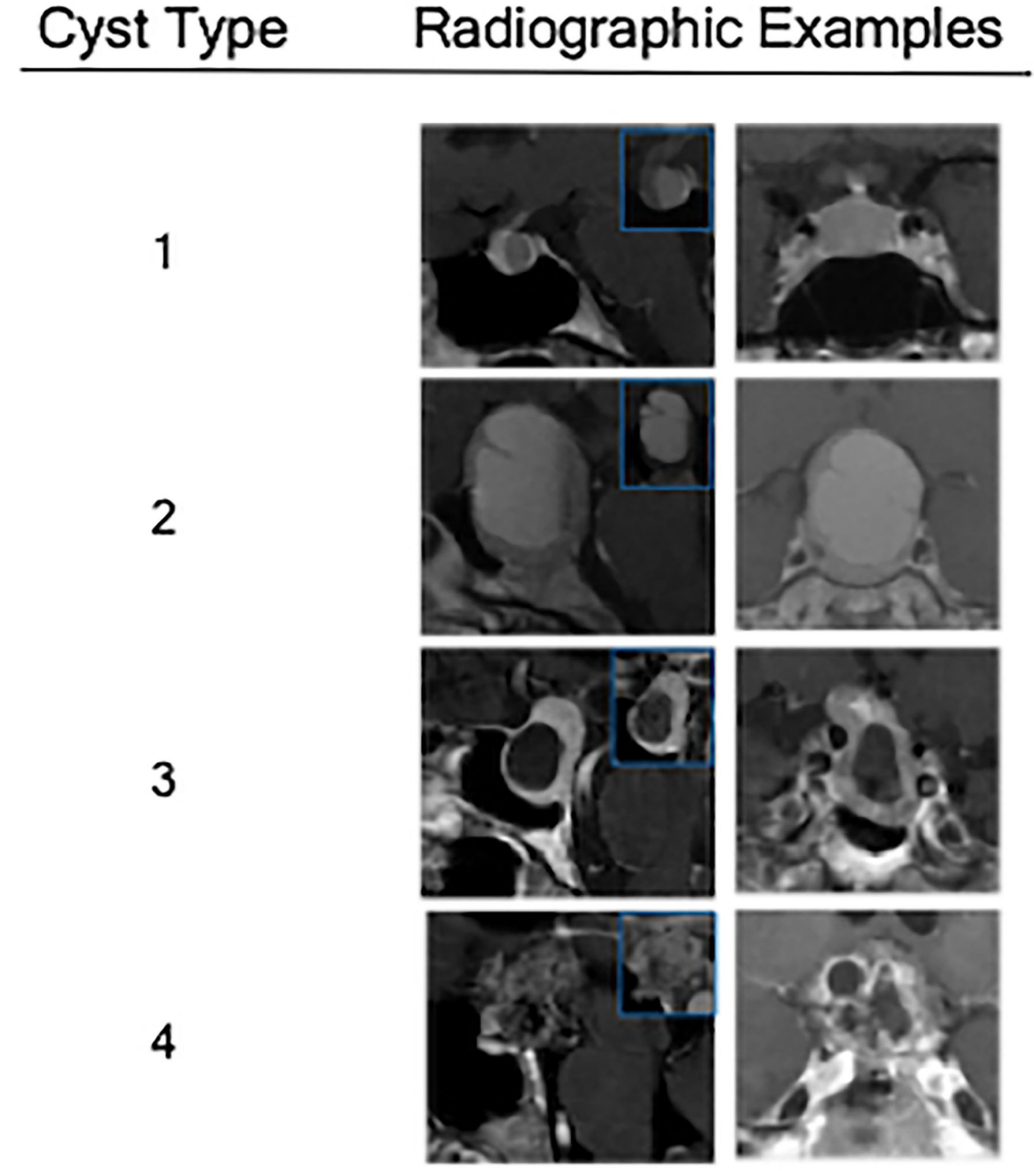
Radiographic examples of each class of cystic sellar masses, with illustrative sagittal post-contrast T1-weighted MRI (left), sagittal pre-contrast T1-weighted MRI (small box), and coronal post-contrast T1-weighted MRI (right).

The primary objective of the study was to estimate the association between cyst type and pathology, especially as it helped distinguish RCC from cystic pituitary adenomas. Cystic lesions were classified by the heterogeneity of the cyst wall/contents and the presence of a solid component on preoperative MRI (**Figure 1**). Type 1 cystic lesions were defined by a well-circumscribed regular border and homogeneous contents with no solid component (i.e. containing cyst contents and cyst wall only). Type 2 cystic lesions were defined by a well-circumscribed but irregular border with heterogenous content/septations and a subtle solid, non-cystic component. Type 3 lesions were defined by a well-circumscribed regular boarder and homogenous contents (like Type 1), but with an obvious solid component. Type 4 lesions were defined by irregular borders with heterogenous contents/septations (like Type 2) with multiple cysts, was predominately solid, and sometimes not well-circumscribed (**Figure 2**). The radiographic classification scheme was consistent and easy to apply. There was unanimous consensus on cyst type for 91% of cases, and 100% agreement between at least 2 of the 3 reviewers (κ=0.86).

### Data Analysis

Student’s t-test, Wilcoxon rank sum tests, Chi-square tests, and Fisher’s exact tests were used for the comparison of continuous and categorical covariates of interest. A purposeful selection approach was used to create a multivariable logistic model. Odds ratios (OR), 95% confidence intervals, and p-values are reported, in addition to receiver operating characteristics (ROC) analysis. All analyses were performed using the SAS/STAT software (Version 9.4, 2013; Cary, NC). Statistical significance was defined as p < 0.05.

## Results

We originally queried an institutional database of 1083 transsphenoidal operations in 982 patients for sellar pathology (757 operations for pituitary adenoma) performed by one of the senior authors (ERL) from April 2008 to January 2020. In total, 205 patients were found to have undergone transsphenoidal surgery for cystic sellar pathology. We classified the lesions of these patients (mean age 42 years, range 16-90 years; 68% women) into the four cyst types (**Table 1**). Type 1 and 2 cysts were the most prevalent (33% and 35%, respectively), with 5% of lesions being classified as Type 3, and 27% as Type 4. In our cohort, RCC and cystic pituitary adenomas comprised the vast majority of cystic lesions (36% and 45%, respectively). Arachnoid cysts (7%) and craniopharyngiomas (6%) were the next most common, with only one patient each having a pathologic diagnosis of chordoma, apoplexy without evident tumor, epidermoid cyst, and colloid cyst. There was a significant association between cyst type and histopathologic diagnosis (p<0.0001; **Table 2**).

**Table 1:** Preoperative patient/lesion characteristics, including patient demographic data, tumor symptoms, serum hormone levels, and radiographic features.

	N (%)
Age, mean (range)	41.7 (16–90)
Sex	
Male	66 (32.2)
Female	139 (67.8)
Obesity	
Yes	70 (34.1)
No	135 (65.9)
History of Other Cysts	
Ovarian, n (% of women)	21 (15.1)
Renal	10 (4.9)
Hepatic	2 (1.0)
Adrenal	0
Pineal Gland	2 (1.0)
PCOS, n (% of women)	5 (3.6)
Inclusion	
Cyst mentioned on radiology report	166 (81.4)
Cyst not mentioned, but seen on T2 image	38 (18.6)
Clinical Presentation	
Headache	132 (64.4)
Visual abnormalities	71 (34.8)
Symptomatic hypopituitarism	69 (33.7)
Symptomatic hyperprolactinemia	51 (25.0)
Apoplexy	4 (1.9)
Hormonal abnormalities	
Hyperprolactinemia	97 (47.3)
GH deficient	13 (6.3)
Acromegaly	4 (2.0)
Cortisol deficient	28 (13.7)
Cushing disease	8 (3.9)
Gonadotropin deficient	42 (20.5)
Thyroid deficient	39 (19.0)
Thyroid excess	2 (1.0)
Pre-operative ADH deficient	10 (4.9)
Cyst Category	
Type 1	68 (33.2)
Type 2	72 (35.1)
Type 3	10 (4.9)
Type 4	55 (26.8)
Lesion Location	
Anterior	125 (61.0)
Posterior	80 (39.0)
Maximum diameter (cm)	
≥ 10mm	151 (73.7)
< 10mm	54 (26.3)
Knosp Score	
0-2	183 (88.8)
3-4	23 (11.2)
Fluid-Fluid level	27 (13.2)

**Table 2:** Cyst category by pathologic diagnosis.

		Pathology*, n (%)			
		Arachnoid Cyst	RCC	Pituitary Adenoma	Craniopharyngioma
Cyst Category	Type 1	8 (11.8)	44 (64.7)	14 (20.6)	1 (1.5)
	Type 2	7 (9.7)	31 (43.1)	29 (40.3)	4 (5.6)
	Type 3	–	3 (30.0)	7 (70.0)	–
	Type 4	–	1 (1.9)	45 (83.3)	8 (14.8)

*p < 0.001. The distribution of the four main pathologies represented by each of the cyst types in our cohort is presented. One colloid cyst (Type 1), one epidermoid cyst (Type 2), and one chordoma (Type 4) are not represented in this table.

The majority of the cystic pituitary adenomas were non-staining tumors (38.7% of adenomas), with prolactin- (31.2%) and ACTH-staining (11.8%) tumors following in prevalence. Approximately 46.2% of pituitary adenomas exhibited evidence of hormone secretion, and the remainder were non-functioning. Among the functioning tumors, 67.4% were prolactinomas, 18.6% presented with Cushing disease and 9.3% were associated with acromegaly. Roughly 30% of patients presented with symptomatic hypopituitarism, and roughly 25% of patients exhibited clinical signs of hyperprolactinemia (**Table 1**).

### Cyst Type and Pathology

Cyst Type 1 lesions (n=68, 33.2% of total) were largely comprised of RCCs (n=44, 65%), followed by cystic pituitary adenomas (n=14, 21%) and arachnoid cysts (n=8, 12%). They were nearly twice as likely to be located posteriorly, and were also, on average, smaller than other cystic lesion types - only 60% had a maximal diameter larger than 1 cm. Type 1 lesions rarely showed radiographic evidence of cavernous sinus invasion (3%). Additionally, clinical evidence of a headache was a common symptom seen in 78% of patients. Finally, patients with Type 1 lesions were younger and more likely to be female (mean age: 37 years; 84% female).

Cyst Type 2 lesions (n=72, 35.1% of total) were primarily either an RCC (n=29, 43%) or a cystic pituitary adenoma (n=28, 41%). These lesions, unlike Type 1 lesions, were located anteriorly in 65% of cases. The higher proportion of cystic pituitary adenomas among this cyst type is in agreement with the anterior predominance. Furthermore, as expected, all adenomas in our cohort, regardless of cyst type, were more likely than RCCs to be anteriorly located (p=0.029). Finally, although 72% of Type 2 cysts were larger than 1 cm in diameter, these cysts rarely exhibited cavernous sinus invasion (1%).

Cyst Type 3 lesions (n=10, 4.9% of total) represented the least prevalent cyst type, and only consisted of RCCs (n=3, 30%) and cystic pituitary adenomas (n=7, 70%). Six of the seven (86%) cystic pituitary adenomas were located anteriorly, and all RCCs were posterior lesions. All Type 3 cysts had a maximal diameter greater than 1 cm, with a mean maximal diameter of 1.9 cm.

Cyst Type 4 lesions (n=55, 26.8% of total) were mostly cystic pituitary adenomas (n=45, 83%). Only 15% of Type 4 cystic lesions were craniopharyngiomas, although, the majority of craniopharyngiomas (62%) fell into this category. These lesions were located anteriorly 82% of the time, and Type 4 lesions were, on average, larger than other cyst types; 87% of Type 4 lesions were larger than 1 cm in diameter. Type 4 lesions were the most likely to show evidence of cavernous sinus invasion on preoperative MRI (35%). They were also most likely to have evidence of pre-operative visual abnormalities (62%). Finally, patients with these complex cysts were, on average, older than other patients, with a mean age of 50 years.

### Differentiating Rathke Cleft Cysts and Cystic Pituitary Adenoma

Type 2 and 4 lesions were significantly more likely to be diagnosed as cystic pituitary adenomas compared to Type 1 lesions (OR: 23.7, 342.6; p=0.033, p<0.0001, respectively; **Table 3**). Those with Type 3 lesions had over a 5-fold increase in odds of being diagnosed as cystic pituitary adenomas as compared with RCC but this did not reach statistical significance, likely due to low power. A fluid-fluid level on preoperative MRI was significantly associated with a cystic pituitary adenoma (OR: 12.7; p=0.023). Obesity (OR: 5.0, p=0.003) and symptomatic hyperprolactinemia (OR: 11.5, p<0.001) were also significantly associated with cystic pituitary adenomas. Interestingly, although lesion relationship to the infundibulum (anterior versus posterior) was significant on univariable analysis, this was not statistically significant in the multivariable model. Additionally, we investigated whether the presence of Cushing disease or elevated serum cortisol level was driving the association between obesity and presence of a pituitary adenoma, however, these confounders were not found to be significant, and obesity was thus shown to be a true independent predictor of adenoma. Similarly, confounding of symptomatic hyperprolactinemia by lesion size was also assessed, but not determined to be significant, and thus, only symptomatic hyperprolactinemia was included in the final model. Our multivariable clinical and radiographic model correctly predicted pathology 83.8% of the time (AUC 0.922; sensitivity 90.2%, specificity 76.0%, positive predictive value 82.2%, negative predictive value 86.4%; **Table 3**).

**Table 3:** Adjusted predictors of cystic pituitary adenomas versus Rathke cleft cysts.

	Odds Ratio	95% Confidence Interval	P-value
Cyst Type 1	REF	REF	REF
Cyst Type 2	23.7	1.3-10.6	**0.033**
Cyst Type 3	5.3	0.9-31.9	0.430
Cyst Type 4	342.6	36.2-999.9	**<0.0001**
Fluid-Fluid Level	12.7	1.4-111.9	**0.023**
Symptomatic Hyperprolactinemia	11.5	3.6-37.1	**<0.0001**
Obese	5.0	1.8-14.2	**0.003**

Odds ratios (OR), 95% confidence intervals (CI), and p-values for predictors of cystic pituitary adenoma compared to Rathke cleft cyst (RCC) in our cohort are presented. Bolded P-values indicate statistical significance.

## Discussion

Differentiating cystic sellar lesions can be challenging given the heterogeneity of cyst appearance on preoperative imaging and the wide array of cystic pathologies that can present in this region. The initial differential strongly impacts the recommended management, especially in incidentally found lesions. Our proposed classification system is transparent and simple to use with a high interrater reliability. It summarizes key imaging features and may be most helpful when used alongside clinical and biochemical findings to steer management and treatment.

### Differentiating Cystic Sellar Lesions

Key differentiating features among cystic lesions include heterogeneity of cyst contents, thickness of the cyst wall, cyst location (anterior versus posterior and midline versus lateral location), globular versus eccentric shape, size, degree of invasiveness, presence of solid component, and presence of intracystic nodule (3–19). We distilled these radiographic characteristics to two main factors: heterogeneity of cyst wall/contents and the presence of solid component. Overall, cyst type was found to be significantly associated with lesion pathology (p<0.0001), with non-neoplastic pathologies (e.g., arachnoid cysts and RCC) falling within Types 1 and 2, and neoplastic lesions (e.g., pituitary adenomas and craniopharyngiomas) categorized more frequently as Types 3 and 4. Interestingly, although the vast majority of Type 2 lesions were larger than 1 cm (72%), these cysts rarely exhibited cavernous sinus invasion (1%). This finding suggest that Type 2 cysts are associated with less invasive pituitary adenomas, which represented 41% (n=28) of type 2 cystic lesions.

### Predictors of Cystic Pituitary Adenoma

This classification scheme has predictive value in a multivariable model differentiating between RCC and cystic pituitary adenoma in the pre-operative setting, correctly predicting the pathology 83.8% of the time. Radiographic features (e.g., cyst Type 2-4 and the presence of fluid-fluid level on MRI) and clinical/biochemical features (e.g., obesity and symptomatic hyperprolactinemia) were significant predictors of cystic adenomas. Our goal was to create a simple and effective tool for the clinician. Further in-depth analysis of multiple MRI and computed tomography sequences should also be employed to further aid in the differentiation of rare, but often misdiagnosed, cystic lesions, like hemorrhagic cystic pituitary adenomas (**Table 4**). Analogous to other reports, we observed that the presence of a fluid-fluid level on preoperative MRI is also predictive of cystic pituitary adenoma as compared with RCC (21, 28). We posit that the prevalence of fluid-fluid level among adenomas may be the result of intracystic hemorrhage from the abnormal vasculature architecture of these cystic lesions. We also redemonstrated that obesity is an independent predictor of pituitary adenoma (29, 30). Body mass index and waist circumference throughout early adulthood have been recently associated with higher risk of pituitary adenoma (31).

**Table 4:** Key preoperative radiographic findings for various cystic sellar pathology.

Cyst Pathology	Key Radiographic Features
Rathke cleft cyst (6, 12, 14, 15, 20)	- T2-hyperintense- Homogenously T2-hypointense/T1-hyperintense with high intrinsic protein content
Hemorrhagic cystic pituitary adenoma (20–24)	- T2-hypointense/T1-hyperintense (can mimic RCC when solid component is lacking)
Arachnoid cyst (6)	- Parallels CSF signal intensity on all MRI sequences
Dermoid cyst (16, 17)	- Follows fat signal intensity on all MRI sequences
Epidermoid cyst (16, 19, 25)	- Follows CSF intensity on T1 and T2, but bright on diffusion-weighted images (DWI)
Craniopharyngiomas (24, 26, 27)	- Contrast enhancement of solid portions on MRI- Intensity of cystic component can be variable depending on proportion of protein, cholesterol, and blood- 90% exhibit calcification on CT

### Surgical Implications for Cystic Prolactinomas

Indications for surgical resection of a pituitary mass include large lesions with mass effect and biochemically active tumors causing clinical hyperpituitarism (2, 32). Symptomatic hyperprolactinemia is the most common form of clinical hyperpituitarism and can be caused by a functional lactotroph tumor (prolactinoma) or stalk effect. Large cysts can produce stalk effect through compression of the infundibulum and suppression of dopaminergic negative feedback to the anterior pituitary gland. Prolactinomas directly secrete prolactin, which can be measured in the serum and used in conjunction with tumor volume to diagnose a prolactinoma with high predictive value (33, 34). Cystic prolactinomas with minimal cellular/solid component, however, may not exhibit marked hyperprolactinemia, thereby mimicking hyperprolactinemia caused by stalk effect. This can lead to misclassification by conventional methods due to the mismatch between size and prolactin level, and, therefore, mimicking stalk effect. In our cohort, we found that the presence of clinical symptoms of hyperprolactinemia was one of the most predictive factors in diagnosing a cystic pituitary adenoma compared to an RCC, independent of serum prolactin level and lesion size. This important finding implies that careful distinction should be made between Type 1 and Type 2 lesions in the presence of a mild elevation of prolactin that may otherwise be attributable to stalk effect.

## Conclusion

This study presents a novel cyst classification scheme for categorizing cystic sellar lesions based on the heterogeneity of the cyst wall/contents and the presence of a solid component on pre-operative MRI. We suggest that cyst type can be used in conjunction with other pre-operative factors to aid in the differentiating RCC from cystic pituitary adenomas, including cystic prolactinomas.

## Data Availability Statement

The raw data supporting the conclusions of this article will be made available by the authors, without undue reservation.

## Author Contributions

ST, MC, and DC were involved in data collection, data analysis, and manuscript writing. XB created figures and provided image formatting. EL provided the idea for the study and the cohort of cases from which the data was derived. All authors were heavily involved in manuscript editing. All authors contributed to the article and approved the submitted version.

## Conflict of Interest

The authors declare that the research was conducted in the absence of any commercial or financial relationships that could be construed as a potential conflict of interest.

## Publisher’s Note

All claims expressed in this article are solely those of the authors and do not necessarily represent those of their affiliated organizations, or those of the publisher, the editors and the reviewers. Any product that may be evaluated in this article, or claim that may be made by its manufacturer, is not guaranteed or endorsed by the publisher.
